# Oleoylethanolamide and Palmitoylethanolamide Enhance IFNβ-Induced Apoptosis in Human Neuroblastoma SH-SY5Y Cells

**DOI:** 10.3390/molecules29071592

**Published:** 2024-04-02

**Authors:** Chiara Camoglio, Jihane Balla, Paola Fadda, Simona Dedoni

**Affiliations:** 1 Department of Biomedical Sciences, Division of Neuroscience and Clinical Pharmacology, University of Cagliari, 09142 Cagliari, Italypaola.fadda@unica.it (P.F.); 2 Neuroscience Institute, National Research Council of Italy (CNR), 09142 Cagliari, Italy

**Keywords:** oleoylethanolamide (OEA), palmitoylethanolamide (PEA), peroxisome proliferator-activated receptor α (PPARα), interferons (IFNs), apoptosis, human neuroblastoma cells

## Abstract

Oleoylethanolamide (OEA) and palmitoylethanolamide (PEA) are endogenous lipids that act as agonists of the peroxisome proliferator-activated receptor α (PPARα). Recently, an interest in the role of these lipids in malignant tumors has emerged. Nevertheless, the effects of OEA and PEA on human neuroblastoma cells are still not documented. Type I interferons (IFNs) are immunomodulatory cytokines endowed with antiviral and anti-proliferative actions and are used in the treatment of various pathologies such as different cancer forms (i.e., non-Hodgkin’s lymphoma, melanoma, leukemia), hepatitis B, hepatitis C, multiple sclerosis, and many others. In this study, we investigated the effect of OEA and PEA on human neuroblastoma SH-SY5Y cells treated with IFNβ. We focused on evaluating cell viability, cell proliferation, and cell signaling. Co-exposure to either OEA or PEA along with IFNβ leads to increased apoptotic cell death marked by the cleavage of caspase 3 and poly-(ADP ribose) polymerase (PARP) alongside a decrease in survivin and IKBα levels. Moreover, we found that OEA and PEA did not affect IFNβ signaling through the JAK-STAT pathway and the STAT1-inducible protein kinase R (PKR). OEA and PEA also increased the phosphorylation of p38 MAP kinase and programmed death-ligand 1 (PD-L1) expression both in full cell lysate and surface membranes. Furthermore, GW6471, a PPARα inhibitor, and the genetic silencing of the receptor were shown to lower PD-L1 and cleaved PARP levels. These results reveal the presence of a novel mechanism, independent of the IFNβ-prompted pathway, by which OEA and PEA can directly impair cell survival, proliferation, and clonogenicity through modulating and potentiating the intrinsic apoptotic pathway in human SH-SY5Y cells.

## 1. Introduction

Type I interferons (IFNs) represent a class of cytokines that are naturally secreted in response to viral infections. They are produced by a variety of cells during the inflammatory response and can, upon activation, induce numerous molecular changes that affect several cellular processes including cell growth and differentiation. These proteins have garnered biomedical interest thanks to their therapeutical action against viral infections, neuro-inflammatory diseases, and tumors including melanoma, hairy cell leukemia, and lymphoma [1,2,3]. Type I IFNs were the first of their type to be produced by recombinant DNA technology as well as the first to be used therapeutically. The first report on their anti-tumoral activity in mice was published almost half a century ago [4]. Although, in recent years, more targeted therapies have been favored in the treatment of tumors, type I IFNs are still highly considered thanks to their ability to induce the expression of more than 200 proteins that are implicated in therapeutic processes, although some are still of unknown activity [5,6]. Consequently, research studies are focusing on unraveling the wide variety of molecular pathways prompted by IFNs to characterize and target them for the treatment of different pathologies. Previously, it was reported that type I IFNs can directly impair the survival of neuroblastoma SH-SY5Y cells by promoting intrinsic apoptosis [5]. Moreover, in different types of cultured malignant cells, type I IFNs were successful in inducing cell death by promoting intracellular events leading to apoptosis [6,7].

PPARα is a member of the peroxisome-proliferator activated receptors (PPARs) family, which includes the PPARα, PPARγ, and PPARβ/δ subtypes that are characterized by distinct tissue distribution. These nuclear receptors promote ligand-dependent transcription of target genes that regulate energy production, lipid metabolism, and inflammation. PPARα is highly expressed in the muscles, heart, kidneys, liver, and small and large intestines [8]. Notably, PPARα agonists are known for their significant role in the treatment of dyslipidemia or metabolic syndromes by reducing plasma triglyceride levels [9] together with the modulation of glucose homeostasis and insulin resistance [10]. The expression of PPARα in peripheral tissue underlies its crucial implication in metabolic pathways that are linked to several conditions including inflammation, cancer, and neurodegeneration [11,12].

Oleoylethanolamide (OEA) and palmitoylethanolamide (PEA) are a class of naturally occurring bioactive lipids derived from saturated and unsaturated fatty acid precursors that display high affinity for the nuclear PPARα receptors [13]. OEA is an endogenous lipid derived from oleic acid, a monounsaturated fatty acid synthesized from membrane glycerophospholipids, while PEA is an endogenous compound belonging to the family of N-acylethanolamines isolated for the first time from purified lipid fractions of soybeans, egg yolk, and peanut meal [13,14,15]. Lipids are a group of complex biomolecules that not only form the structural basis of biological membranes but also function as signaling molecules and as a source of energy. In recent years, lipids have emerged as central players in a complex network that modulates cellular and molecular actions associated with different physiopathological states [14,15]. While the effect of PEA on B16 melanoma, MCF-7 breast, colon HCT116, and astrocytoma cells has been documented, little is known about the impact of OEA and PEA exposure on neuroblastoma [12,16,17]. The focus of our research is mainly directed towards elucidating any possible overlapping signaling pathways that may be activated by these two lipids in the human neuroblastoma SH-SY5Y cell line when present simultaneously with IFNβ in a pharmacological combination, thereby suggesting a possible contributing role of OEA and PEA in the development of alternative therapeutic strategies.

## 2. Results

### 2.1. OEA and PEA Potentiate the Effect of IFNβ on Cell Viability in SH-SY5Y Cells

**Figure 1:**
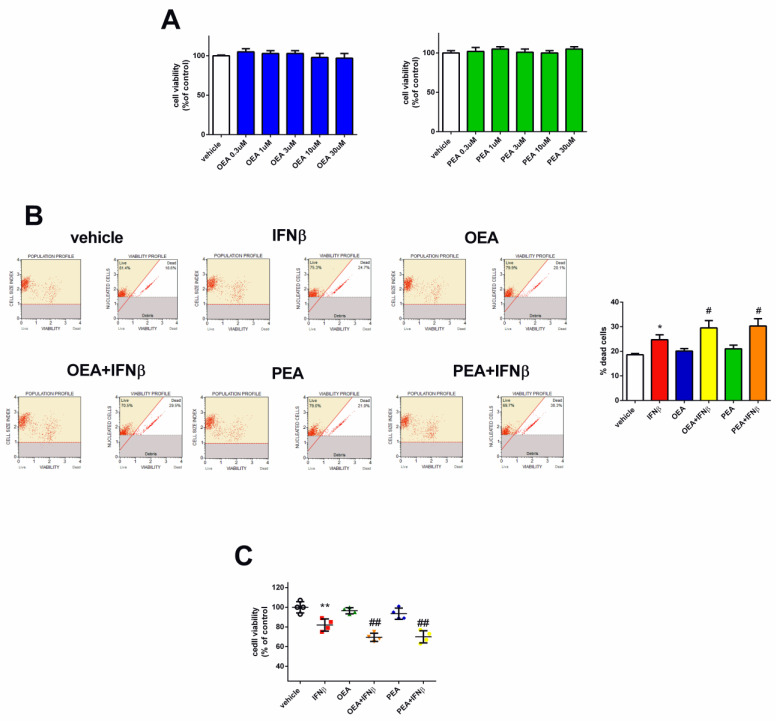
OEA, PEA, and IFNβ treatment effect on SH-SY5Y cell viability. Cells were treated for 24 h with vehicle, OEA, or PEA. Cell viability was then assessed by the MTT assay (**A**). Cells were pre-treated with OEA and PEA (3 µM–6 h) and then stimulated with IFNβ (5 ng/mL–24 h). The viability was measured by the Muse^®^ Viability kit. Values are reported as the percentage of dead cells and are represented as the mean ± SEM of four experiments. * *p* < 0.05 versus vehicle and ^#^ *p* < 0.05 versus IFNβ (**B**). SH-SY5Y cells were pre-treated with OEA and PEA (3 µM–6 h) and then stimulated with IFNβ (5 ng/mL–24 h) and cell viability was assessed by a luminescence assay. The values are expressed as percentage of control (vehicle) and the results are represented as the mean ± SEM of four independent experiments. ** *p* < 0.01 versus vehicle and *^##^ p* < 0.01 versus IFNβ (**C**).

In order to depict the role of OEA and PEA in SH-SY5Y cells, the potential cytotoxicity of these two compounds was first evaluated by an MTT assay. Based on prior studies investigating the efficacy of OEA and PEA on colon cancer cells and mouse neuroblastoma N1E-115 cells [12,18], we utilized concentrations ranging from 0.3 to 30 µM for the treatment of SH-SY5Y cells for a period of 24 h. As reported in Figure 1A, cell viability was not affected by both lipids at all concentrations. Next, we analyzed the effects of these two PPARα agonists together with IFNβ. Since there were no differences in the effect of the different concentrations of the two lipids on cell viability, we opted for a 3 µM concentration as it is consistent with its prior application in another neuroblastoma cell line, as reported by Hamatiaux and colleagues [18]. In addition, and given the nuclear localization of PPARα, a 6 h pre-treatment period with either OEA or PEA was carefully undertaken to ensure optimal access of these lipids to their receptor. Hence, SH-SY5Y cells were pre-treated with OEA and PEA (3 µM - 6 h) and then stimulated with IFNβ (5 ng/mL −24 h). Cell viability was assessed by the Muse™ Cell Analyzer (Millipore Corporation, Merck Life Sciences, Darmstadt, Germany)). OEA and PEA did not alter cell viability compared to vehicle-treated cells (Figure 1B) while the associations of OEA + IFNβ and PEA + IFNβ increased cell death in comparison to IFNβ (*p* < 0.05). To further validate the cell viability results, the RealTime-Glo™ MT assay involving engineered luciferase was performed. As shown in Figure 1C, OEA + IFNβ and PEA + IFNβ reduced viability compared to IFNβ (*p* < 0.05).

### 2.2. OEA and PEA Exacerbate the Effect of IFNβ on Cell Proliferation and Clonogenicity in SH-SY5Y Cells

**Figure 2:**
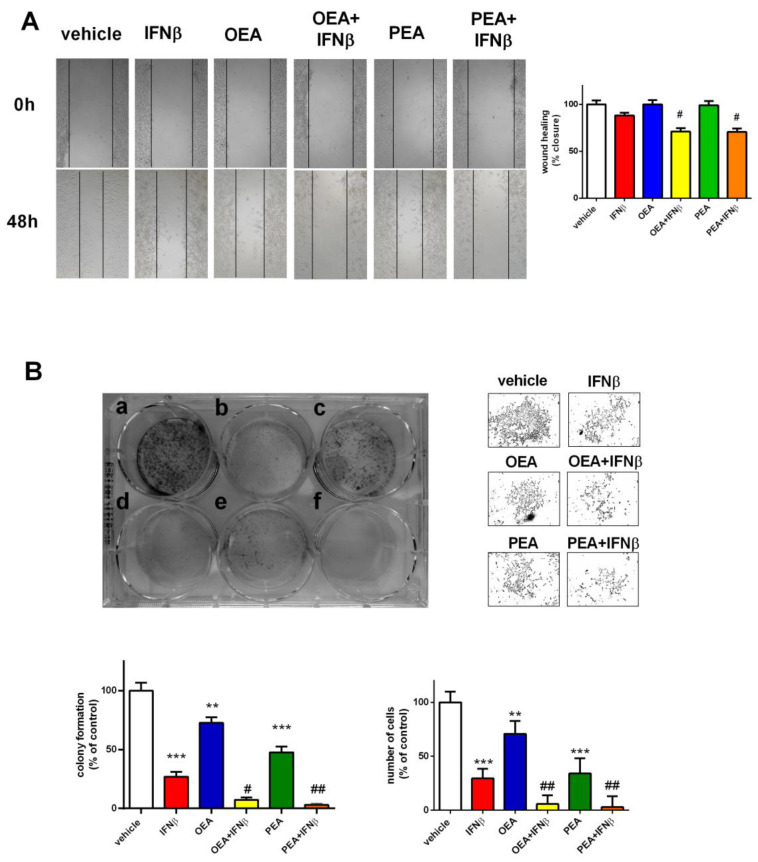
OEA, PEA, and IFNβ effect on cell proliferation and clonogenicity. Cells were treated for 48 h with the vehicle, OEA or PEA (3 µM), and IFNβ (5 ng/mL). Representative images show the scratch wound healing assay performed on treated SH-SY5Y cells. The percentage of wound closure in the scratch assay was measured. Values are reported as the mean ± SEM of four experiments. ^#^ *p* < 0.05 versus IFNβ. Magnitude 10× (**A**). Cells were seeded at a cell density of 200 cells/well in a six-well plate for the clonogenic assay and were exposed to (**a**) vehicle; (**b**) IFNβ 5 ng/mL; (**c**) OEA 3 µM; (**d**) OEA + IFNβ; (**e**) PEA 3 µM; or (**f**) PEA + IFNβ. Cells were stained with crystal violet to visualize colonies formed by 50 or more cells. Representative pictures of cell density were taken under a microscope after treatment. The number of formed colonies and the number of cells present in each well were quantified and are represented above as percentage of control (vehicle). ** *p* < 0.01 and *** *p* < 0.001 versus vehicle-treated cells; ^#^ *p* < 0.05 and ^##^ *p* < 0.01 versus IFNβ. Values are reported as the mean ± SEM of four experiments (**B**).

To further explore the effects mediated by OEA and PEA on IFNβ-stimulated cells, the proliferation assay was assessed by the scratch wound healing test through the measurement of wound closure. As reported in Figure 2A, no difference was detected in the wound healing between cells exposed to OEA or PEA alone in comparison to vehicle-treated cells. On the other hand, the co-exposure of either OEA or PEA along with IFNβ further decreased wound healing potential (*p* < 0.05). Moreover, SH-SY5Y cells were seeded at clonogenic density in six-well plates and were allowed to grow for 10 days. As shown in Figure 2B, OEA and PEA alone were able to reduce colony efficiency by 28% (*p* < 0.01) and 53% (*p* < 0.001), respectively, in comparison to vehicle-treated cells. Additionally, co-exposure to OEA or PEA and IFNβ reduced the number of colonies by 20% (*p* < 0.05) and 24% (*p* < 0.01), respectively, when compared to IFNβ alone. Furthermore, the effect of co-exposure on cell proliferation was further substantiated by the significant decrease reported from cell quantification (*p* < 0.01, OEA + IFNβ, and PEA + IFNβ versus IFNβ) (Figure 2B).

### 2.3. OEA and PEA Potentiate Apoptosis in IFNβ-Treated Cells

**Figure 3:**
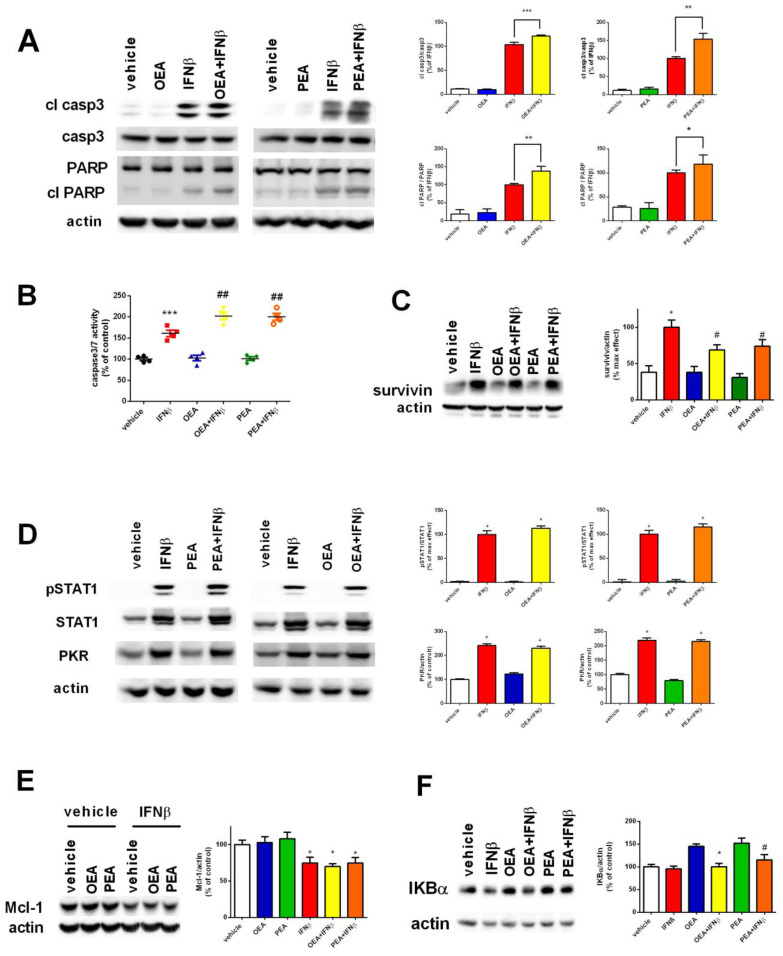
Assessment of intrinsic apoptosis after exposure to OEA, PEA, and IFNβ. Cells were pre-treated with OEA and PEA (3 µM - 6 h) and then stimulated with IFNβ (5 ng/mL - 24 h). Cell lysates were analyzed for cleaved caspase 3 and cleaved PARP by Western blot. The ratio between the cleaved protein and its total form was measured. The values are expressed as a percentage of IFNβ. * *p* < 0.05, ** *p* < 0.01 and *** *p* < 0.001 versus IFNβ (**A**). The caspase 3/7 activity was measured by a luminescence assay. The values are expressed as percentage of control (vehicle). *** *p* < 0.001 versus vehicle and ^##^ *p* < 0.01 versus IFNβ. The results are represented as mean ± SEM of four independent experiments (**B**). Cells were treated as in (**A**). The cell lysates were analyzed for survivin. Values are expressed as percentage of control maximal effect. * *p* < 0.05 versus vehicle and ^#^ *p* < 0.05 versus IFNβ (**C**). Cells were treated as mentioned in (**A**). Cell lysates were used to assess the activation of the JAK-STAT pathway and PKR induction. The values are expressed as a percentage of maximal effect. * *p* < 0.05 versus vehicle (**D**). Cells were treated as in (**A**). The cell lysates were analyzed for Mcl-1. The values are expressed as a percentage of control (vehicle). * *p* < 0.05 versus vehicle (**E**). Cell lysates were analyzed for IKBα. The values are expressed as a percentage of control (vehicle). * *p* < 0.05 and ^#^ *p* < 0.05 versus OEA and PEA, respectively. The results are represented as the mean ± SEM of four independent experiments (**F**).

Previously, it was reported that IFNβ can activate the intrinsic apoptotic pathway in SH-SY5Y cells [5]. A 12 h exposure to IFNβ was sufficient to activate caspase 3/7; however, the co-treatment with OEA or PEA was not sufficient to induce any potentiation in this time frame (Figure S1). Next, we proceeded to investigate apoptosis at 24 h as previously conducted on SH-SY5Y cells [5]. Exposure to OEA or PEA alone does not promote any increase in the cleavage of these two proteins, nevertheless, cells co-treated with IFNβ (24 h) showed an increase in the cleaved form of caspase 3 (19 and 17 KDa) (*p* < 0.001, OEA + IFNβ and *p* < 0.01, PEA + IFNβ versus IFNβ) as well as an increase in cleaved PARP (*p* < 0.01, OEA + IFNβ and *p* < 0.05, PEA + IFNβ versus IFNβ) (Figure 3A). This was also confirmed by the measurement of caspase 3/7 activity (Figure 3B). Next, we investigated survivin, a protein belonging to the inhibitors of apoptosis (IAP) family that can be modulated by IFNs and can affect apoptosis. Exposure to IFNβ was able to increase its levels, as shown in Figure 3C (*p* < 0.05). However, co-exposure with OEA or PEA, as previously reported in the literature, significantly decreased survivin (*p* < 0.05, OEA + IFNβ or PEA + IFNβ versus IFNβ) [19]. Reports have demonstrated that type I IFNs can induce apoptosis in SH-SY5Y cells through multiple mechanisms involving JAK-STAT signaling and PKR induction [5]. Based on these findings, we examined whether the increased activation of the apoptotic pathway mediated by OEA and PEA might be caused by a potentiation of the IFNβ-stimulated JAK-STAT pathway and/or by an improvement in protein induction. By combining the treatment of OEA or PEA and IFNβ, STAT1 phosphorylation, total STAT1, and PKR induction were all assessed using the Western blot assay. Our results revealed that continuous exposure to OEA or PEA along with IFNβ did not further affect STAT1 phosphorylation, STAT1, or PKR in comparison to IFNβ-treated samples (Figure 3D). To elucidate further the signaling pathways prompted by OEA and PEA in SH-SY5Y cells, we investigated the expression of other different proteins. No changes in the expression of pro- or anti-apoptotic proteins such as Bax and Bcl2 were observed. Surprisingly, Mcl-1, which belongs to the Bcl2 family and is known to be involved in the control of cell survival by preventing the activation of apoptosis, was found to be significantly reduced in SH-SY5Y cells treated with IFNβ (*p* < 0.05). In contrast, no influence was appointed by co-treatment with OEA or PEA (Figure 3E). According to previous reports, persistent OEA and PEA treatment in SH-SY5Y cells may elevate IKBα levels. Additionally, PEA has been proven to prevent IKBα degradation [20,21]. Our results show that the increase in IKBα levels normally elicited by OEA and PEA treatment was hindered by co-exposure to IFNβ, therefore indicating that the anti-inflammatory effects of OEA and PEA were insufficient to counteract IFNβ’s inflammatory role as proven by the basal levels of IKBα reported by the cytokine in Figure 3F (*p* < 0.05 versus OEA or PEA).

### 2.4. OEA and PEA Amplify the IFNβ-Mediated Increase in PD-L1 Expression and Phosphorylated p38 MAPK

**Figure 4:**
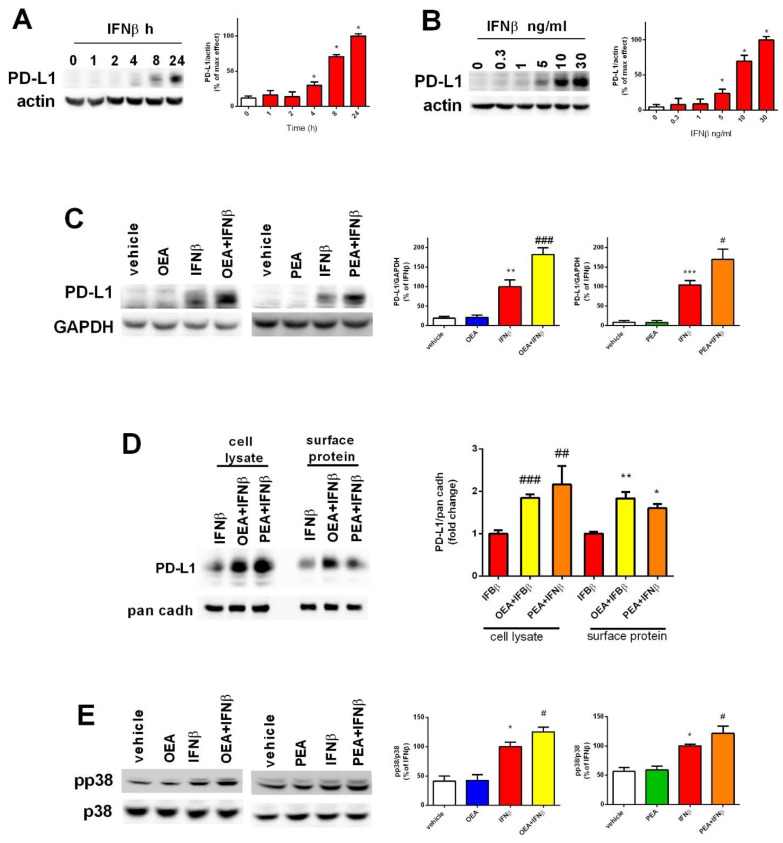
Involvement of PD-L1 and p38 MAPK in the effects of OEA, PEA, and IFNβ treatment on SH-SY5Y cells. Cells were treated with IFNβ for different lengths of times (**A**) and at different concentrations (**B**) to assess the effect of time and dose exposure to IFNβ on the levels of the programmed death-ligand 1 (PD-L1). * *p* < 0.05 versus vehicle. The values are expressed as percentage of IFNβ and are represented as the mean ± SEM of four independent experiments (**A**,**B**). Cells were treated as mentioned in Figure 3A and cell lysates were analyzed for PD-L1. The values are expressed as percentage of IFNβ. ** *p* < 0.01 and *** *p* < 0.001 versus vehicle; ^#^ *p* < 0.05 and ^###^ *p* < 0.001 versus IFNβ. The results are represented as the mean ± SEM of four independent experiments (**C**). Cells were treated as reported in Figure 3A. The total cell extract (cell lysate) and biotinylated proteins (surface protein) were analyzed for PD-L1 by Western blot. The levels of PD-L1 in whole cell lysate and cell surface were normalized to pan cadherin (pan cadh), a plasma membrane marker. * *p* < 0.05 and ** *p* < 0.01 in surface protein versus IFNβ; ^##^ *p* < 0.01 and ^###^ *p* < 0.001 in the cell lysate versus IFNβ. Values are represented as the mean ± SEM of three independent experiments (**D**). Cells were treated as in Figure 3A, and cell lysates were analyzed for the phosphorylated and total p38 protein. * *p* < 0.05 versus vehicle; ^#^ *p* < 0.05 versus IFNβ (**E**).

IFNs are known to upregulate the expression of the programmed death-ligand 1 (PD-L1), a type 1 transmembrane glycoprotein. PD-L1 is the ligand of the programmed cell death-1 (PD-1) receptor. IFNs can induce its expression in tumors, leading to immune evasion and the enhancement of cancer cell survival [22,23]. Indeed, Figure 4A,B indicates that PD-L1 rose in a time- and concentration-dependent manner following IFNβ treatment, reaching its peak levels at 24 h and showing a detectable band at a concentration of 5 ng/mL. Most importantly, results show that co-treatment using OEA or PEA and IFNβ increases PD-L1 levels in whole cell lysate as reported in Figure 4C. Neither compound displayed a reactive band in the Western blot assay when used alone (*p* < 0.001, OEA + IFNβ and *p* < 0.05, PEA + IFNβ versus IFNβ) (Figure 4C). To provide more insight about PD-L1 after OEA or PEA and IFNβ treatment, the cell surface expression of the latter was examined. As shown in Figure 4D, exposure to IFNβ alone increased the cell surface levels of PD-L1. This expression was further increased by the co-treatment with either OEA or PEA by 1.8- and 1.6-fold, respectively, in comparison to IFNβ-treated cells (*p* < 0.01, OEA + IFNβ and *p* < 0.05, PEA + IFNβ). The existence of a causal link between IFNβ and an anti-apoptotic outcome mediated through the activation of the p38 MAPK, a collateral signaling pathway that opposes the activity of programmed cell death, was formerly reported [24]. As shown in Figure 4E, p38 phosphorylation significantly increased in samples treated with OEA and PEA in the presence of IFNβ compared to IFNβ (*p* < 0.05 versus IFNβ). These two mechanisms, PD-L1 and the p38 MAPK pathway, might explain how OEA and PEA enable cells to respond and adjust to the apoptotic insult triggered by IFNβ. Both mechanisms may form a safety system that compensates for the absence of IKBα intervention that is normally elicited by OEA and PEA treatment but has been hindered by the potent effect of IFNβ stimulation.

### 2.5. PPARα Contributes to OEA and PEA Enhancement of PD-L1 and IFNβ-Induced Apoptosis

**Figure 5:**
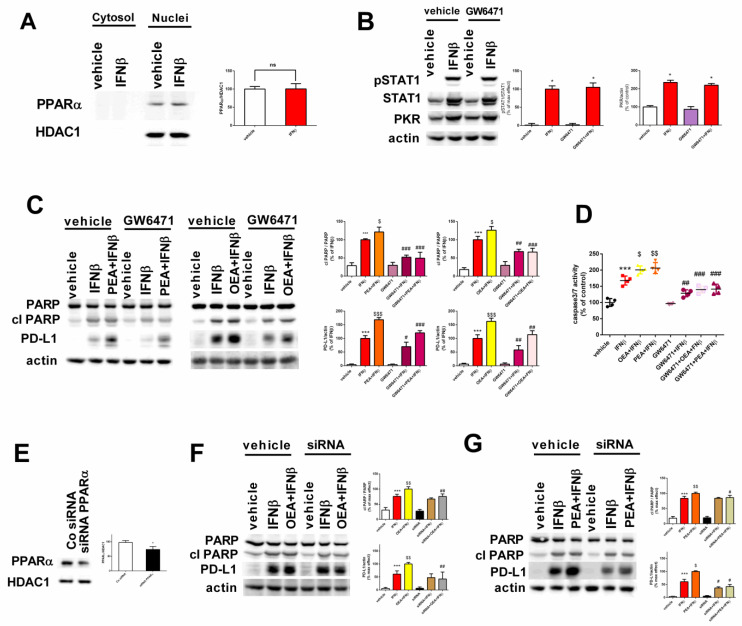
Implication of the PPARα receptor in the effects mediated by OEA, PEA, and IFNβ. Nuclear extracts were prepared and analyzed for PPARα receptor levels after IFNβ exposure. Values are represented as the mean ± SEM of four experiments; ns = not significant (**A**). Cell lysates were analyzed for STAT1 phosphorylation, STAT1, and PKR after treatment with IFNβ alone for 24 h in the presence and absence of GW6471 (6 μM). * *p* < 0.05 versus vehicle (**B**). Cells were pre-treated with 6 μM GW6471 (1 h) and were then exposed for 6 h to OEA or PEA, followed by IFNβ (24 h). Cell lysates were analyzed for PD-L1 and cleaved PARP (cl PARP). *** *p* < 0.001 versus vehicle; ^$^ *p* < 0.05 and ^$$$^ *p* < 0.001 versus IFNβ; ^#^ *p* < 0.05, ^##^ *p* < 0.01, and ^###^ *p* < 0.001 versus the corresponding value in control-treated cells (**C**). Cells were treated as mentioned before and caspase 3/7 activity was determined by a luminescence assay. *** *p* < 0.001 versus vehicle; ^$^ *p* < 0.05 and ^$$^ *p* < 0.01 versus IFNβ; ^##^ *p* < 0.01 and ^###^ *p* < 0.001 versus the corresponding value in control-treated cells (**D**). SH-SY5Y cells were transfected with either control siRNA or PPARα siRNA. * *p* < 0.05 versus vehicle-treated cells (**E**). Cells transfected with either control siRNA or PPARα siRNA were incubated for 24 h with either vehicle, IFNβ, or co-treated with IFNβ and OEA or PEA. Cell lysates were analyzed for cleaved PARP and PD-L1. *** *p* < 0.001 versus vehicle; ^$^ *p* < 0.05 and ^$$^ *p* < 0.01 versus IFNβ; ^#^ *p* < 0.05, ^##^ *p* < 0.01 versus the corresponding value in control siRNA-treated cells. Values are the mean ± SEM of four independent experiments (**F**,**G**).

IFN-stimulated genes are expressed in response to IFN stimulation and are known to be capable of mediating biological and therapeutical effects by inducing the production of a plethora of proteins in cells of different origins [25]. To assess if a 24 h exposure to IFNβ can modify the expression of PPARα, nuclei were extracted from SH-SY5Y cells after IFNβ treatment. As shown in Figure 5A, cell exposure to IFNβ did not affect PPARα receptor levels.

Additionally, GW6471, a PPARα receptor inhibitor, was used to evaluate this receptor’s involvement. We first assessed STAT1 phosphorylation, STAT1, and PKR induction to reveal any possible interaction with IFNβ signaling. Our results report no interference as shown in Figure 5B. Next, we investigated the effect of OEA, PEA, and IFNβ on the levels of cleaved PARP and PD-L1 in the presence of the PPARα inhibitor. GW6471 reduced PARP cleavage (*p* < 0.001, GW6471 + OEA + IFNβ versus OEA + IFNβ and GW6471 + PEA + IFNβ versus PEA + IFNβ) and PD-L1 levels in cells co-exposed to OEA or PEA and IFNβ (*p* < 0.01 in GW6471 + OEA + IFNβ and *p* < 0.001 in GW6471 + PEA + IFNβ versus the corresponding control value in vehicle-treated cells) (Figure 5C). Moreover, the caspase 3/7 activity in SH-SY5Y cells confirmed the results reported above, as seen in Figure 5D.

To further clarify whether the modulation of PD-L1 levels and PARP cleavage were mediated by the PPARα receptor, SH-SY5Y cells were transfected with a specific siRNA targeting PPARα (Figure 5E). The transfection suppressed the increase in PD-L1 protein levels in siRNA + OEA + IFNβ and siRNA + PEA + IFNβ versus the corresponding value in control siRNA-treated cells (*p* < 0.01, versus OEA + IFNβ and *p* < 0.05, versus PEA + IFNβ, respectively) and reduced PARP cleavage in siRNA + OEA + IFNβ and in siRNA + PEA + IFNβ versus the corresponding value in control siRNA-treated cells (*p* < 0.01 and *p* < 0.05, respectively), suggesting that the OEA and PEA might enhance IFNβ-mediated apoptosis partially through the PPARα receptor (Figure 5F,G).

## 3. Discussion

In this study, we investigated the effects of OEA and PEA, in association with IFNβ, on human neuroblastoma SH-SY5Y cells. We also focused on elucidating the intracellular mechanisms that underlie the observed impairments in cell viability and proliferation to provide key proof of the detrimental effects mediated by these two lipids in SH-SY5Y neuroblastoma cells. The enhanced synthesis and absorption of lipids are known to contribute to the rapid growth of cells, the onset of inflammation, and tumorigenesis [14]. As a bioactive lipid, PEA’s involvement has been previously documented in different cancer cell lines of different origins, such as B16 melanoma, MCF-7 breast, and HCT116 colon cells [12,16,17,26]. So far, little is known about how OEA and PEA may affect human neuroblastoma SH-SY5Y cells. Initially, the toxicity of OEA and PEA in SH-SY5Y cells was evaluated using a cell viability MTT assay. After 24 h of exposure to OEA and PEA at concentrations ranging from 0.3 to 30 µM, SH-SY5Y cells showed no signs of cellular suffering. In contrast, Pagano and colleagues reported a reduction in the proliferation of colon cancer cells after exposure to PEA at concentrations ranging from 3 μM to 30 μM, while treatment at 1 μM showed no significant effect [12]. In addition, the exposure of N1E-115 mouse neuroblastoma cells to OEA and PEA at 1 μM and 5 μM, respectively, was able to reduce cell viability [18]. These findings demonstrate how the sensitivity and the response to OEA and PEA treatment can change depending on the concentration, the treatment duration, and the type of the cancer cell line itself (i.e., colon cancer cells, mouse neuroblastoma cells) [12,18] as well as the cellular microenvironment, which can also be very different from what could potentially be observed in vivo as the data reported above all refer to in vitro investigations. The molecular and phenotypic heterogeneity of tumors and, consequently, their susceptibility to various forms of treatment must also be considered along with the nature of the patient’s physiological state dictated by age, inflammation, immune system response, pathologies, and so on.

Interestingly, our results show that OEA and PEA can drastically lower cell viability when co-exposed with IFNβ in SH-SY5Y cells. The same effect was also observed through the wound healing assay in which the proliferation was negatively affected. Furthermore, the co-treatment with either OEA or PEA and IFNβ for 10 days was able to decrease colony formation in the clonogenic test, an approach that is regularly used in in vitro settings to assess clonogenicity. These results are in line with previous findings demonstrating that PEA inhibited tumor cell proliferation and migration through the PPARα receptor in colon cancer cells [12]. Moreover, our results also show that treatment with OEA and PEA alone can hinder cell proliferation and colony formation in comparison to the control group, showing that these two lipids are not harmful to SH-SY5Y cells after a 24 h treatment but do, however, exert detrimental effects at longer periods of exposure.

To determine the intracellular events that underlie the enhanced impairment in both cell viability and proliferation, we investigated the intrinsic apoptotic pathway, a mechanism that has been previously recognized to prompt IFNβ’s detrimental effect in SH-SY5Y cells [5]. Our findings showed that the co-administration of the two lipids with IFNβ is more effective than IFNβ alone at promoting the cleavage of caspase 3 and PARP, therefore indicating the potentiation of intrinsic apoptotic cell death without the amplification of the main IFNβ signaling cascade. Future research is necessary to determine how these compounds can affect other neuroblastoma cell lines. A study reported that an engraftment of human IMR32 neuroblastoma cells in vivo was responsive and restricted by type I IFN treatment [27]. Nevertheless, more investigations should be carried out while taking into account neuroblastoma cell lines that display differences in genetic amplification, p53 mutation or overexpression, and ALK receptor constitutive active involvement. Previous research has shown that IFNβ signaling through STAT1 affects survivin, an inhibitor of apoptosis (IAP) protein, whose levels may be modulated by the JAK-STAT system [28,29,30]. Our investigation found that IFNβ increased survivin levels, but co-exposure to OEA and PEA impaired this effect. This result might explain the lipid-mediated enhancement of IFNβ-induced apoptosis, as the decrease in survivin levels may represent another mechanism that contributes to the increase in cell death, along with the enhancement of the cleavage of caspase 3 and PARP. A study by Wang and colleagues reported that the stimulation of PPARγ induces cell death through the downregulation of survivin expression and the increase in caspase 3 activity in colorectal cancer cells [19]. These findings are in line with our results that demonstrate both a decrease in survivin and an increase in caspase 3 cleavage and caspase 3/7 activity upon co-treatment with OEA or PEA. Furthermore, OEA and PEA actions are inherently STAT1-independent as demonstrated by the fact that these two lipids are unable to potentiate the JAK-STAT pathway. IFNβ, OEA, and PEA modulate survivin through distinct mechanisms, explaining their different effects when used alone or in combination. However, it is crucial to emphasize that survivin expression undergoes regulation through several other pathways, and many variables, including alternative signaling cascades, might have a significant impact on this protein. The intricate processes by which STAT1 as well as PPARα regulate survivin expression require more in-depth investigations.

Studies have also reported that prolonged exposure to OEA and PEA alone elevates IKBα levels in SH-SY5Y cells [20,21]. Nevertheless, our findings suggest that co-treatment with IFNβ disrupts this enhancement, indicating that IKBα failed at counteracting the cellular damage elicited by this pro-inflammatory cytokine. Mcl-1 is another anti-apoptotic protein from the Bcl-2 family that regulates cell survival, affecting the balance between cell viability and death. Similarly, IFNγ has been reported to downregulate Mcl-1 expression, leading to the promotion of apoptosis [31]. In addition, the suggested association of IFNs with apoptosis can be potentially traced to the downregulation of anti-apoptotic proteins such as Mcl-1 [32]. In our experiments, IFNβ alone decreased Mcl-1 levels. This decrease was not further modulated by co-exposure with either OEA or PEA. The totality of these findings suggests a decrease in the anti-apoptotic cell response, which may lead to increased cell death by IFNβ in combination with OEA or PEA. Most likely, the amalgamation between the reduction in Mcl-1 by IFNβ along with the downregulating action of OEA and PEA on survivin expression and the reduction in IKBα levels by IFNs compromise cellular homeostasis and render cells more susceptible to apoptosis.

IFNs can also induce PD-L1, a ligand of the PD-1 receptor that works as an immune-inhibiting checkpoint, leading to immune evasion and inhibiting antitumoral immune responses through the JAK-STAT pathway [22,23]. Our results demonstrate, for the first time, an increase in the levels of PD-L1 in both cell lysate and cell membranes of SH-SY5Y cells co-treated with OEA or PEA and IFNβ. PPARγ agonists have been shown to increase PD-L1 protein expression in human gastrointestinal and colorectal cancer cell lines [33]. In our investigations, OEA and PEA alone were unable to induce any variation in PD-L1 levels. We also formerly reported that during the induction of apoptosis in SH-SY5Y neuroblastoma cells, IFNβ triggers a collateral signaling pathway mediated by p38 MAPK that opposes the activity of programmed cell death to counteract the cell damage induced by this cytokine [24]. Our current work reports a slight but significant increase in p38 MAPK phosphorylation in samples treated with IFNβ and OEA or PEA concurrently. Although the contribution of these two distinct proteins, p38 MAPK phosphorylation, and PD-L1 might point to a possible synergism attempting to block the apoptotic damage elicited by IFNβ, our results prove that this effect is insufficient in shielding SH-SY5Y cells from the damage brought on by concomitant treatment as suggested by the decrease in cell survival and proliferation as well as survivin, Mcl-1, and IKBα levels as mentioned above. On the other hand, studies have shown that OEA and oleic acid decrease PD-L1 expression induced by IFNγ in human lung carcinoma cells [34]. The varied results obtained by Yamagata and colleagues may be due to the different cellular lines and the different kinds of IFNs employed in their study. Previous reports demonstrate that PD-L1 likewise increases in other neuroblastoma cells apart from SH-SY5Y by IFNγ [35], indicating that different human neuroblastoma cell lines are responsive to IFNs when it comes to PD-L1 rise; however, future studies are necessary to determine if the OEA- and PEA-mediated potentiation of IFNβ-induced PD-L1 levels is present in other neuroblastoma cell lines. Increased expression of PD-L1 can also represent an important target for cancer therapy. Indeed, several treatments that target PD-L1/PD-1 interaction have been recently approved for cancer therapy, including monoclonal antibodies and immune checkpoint inhibitors, since tumors displaying a higher expression of PD-L1 appear to be more sensitive to such treatments [36].

To assess the involvement of the PPARα receptor in the mediation of the observed effects elicited by treatment with OEA, PEA, and IFNβ, we first investigated the expression of the latter after IFNβ exposure and we found no significant difference. Furthermore, to address the direct role of this receptor in OEA- and PEA-induced apoptosis as well as PD-L1 potentiation, we used a specific PPARα receptor antagonist, the compound GW6471. This inhibitor partially blocked PD-L1 potentiation and reduced the PARP cleavage elicited by both IFNβ alone and by the co-treatment with OEA and PEA, while no effect in vehicle-pretreated cells or GW6471 was detected. These findings are further corroborated by the measurement of caspase 3/7 activity which shows the implication of PPARα in the mediation of the effects prompted by the different treatments. Additionally, the fact that GW6471 did not affect the signaling pathway of IFNβ reveals the PPARα inhibitor’s selectivity.

In favor of gaining more insight into PPARα involvement, this receptor was silenced by cell transfection with a specific PPARα siRNA. The experiment demonstrated that in PPARα siRNA-treated samples, IFNβ alone or in combination with OEA or PEA reduced PD-L1 and, to a lesser extent, the potentiation of PARP cleavage. The results suggest that the PPARα receptor is an active contributing element to the increase in apoptosis and PD-L1 by the co-treatment of OEA, PEA, and IFNβ. However, current data leave the possibility for other cellular mechanisms that may participate in OEA- and PEA-mediated apoptosis with IFNβ. Further research is needed to evaluate other collateral pathways that can intervene in OEA and PEA signaling in the SH-SY5Y neuroblastoma cell line.

## 4. Materials and Methods

### 4.1. Materials

OEA and GW6471 were purchased from Tocris (Abingdon, UK) and PEA was obtained from Abcam (Cambridge, UK). rh IFN-beta 1b was from ImmunoTolls (Friesoythe, Germany). Muse™ reagents were obtained from Luminex Corporation (Austin, TX, USA). Caspase-Glo^®^ 3/7 and the RealTime-Glo™ MT Cell Viability assay kit were purchased from Promega (Madison, WI, USA).

### 4.2. Cell Culture

Human neuroblastoma cell line SH-SY5Y was obtained from the European Collection of Authenticated Cell Cultures (ECACC) (Salisbury, UK). The cell line was authenticated by the vendors. SH-SY5Y cells were grown in Ham’s F12/MEM medium (1:1) (Sigma-Aldrich, St. Louis, MO, USA) containing 2 mM L-glutamine (Sigma-Aldrich) and 1% non-essential amino acids (NEAA) (Sigma-Aldrich). The cells were maintained at 37 °C in a humidified atmosphere of 5% CO2 in air. Cells were split every 72 h using 0.25% trypsin/EDTA (Sigma-Aldrich). After resuscitation, the cells were used for no more than 15 passages. The cells were checked for mycoplasma by using the MycoFluor Mycoplasma Detection kit (Invitrogen-Life Technologies, Monza, Italy).

### 4.3. Cell Treatment and Cell Lysate Preparation

Unless otherwise specified, neuroblastoma cells were washed and incubated in a medium containing no FCS. The cells were treated with the test agents as indicated in the text and were maintained at constant temperature and humidity conditions as mentioned earlier. To prepare cell lysates, cells were first washed with PBS and then scraped into an ice-cold lysis buffer (RIPA buffer), supplemented with 1 mM phenylmethylsulphonyl fluoride (PMSF), 0.5% phosphatase inhibitor cocktail 3, and 1% protease inhibitor cocktail (Sigma-Aldrich). The samples were sonicated for 5 s and cell extract aliquots were taken for protein analysis by using the Bio-Rad protein assay (Bio-Rad Lab, Hercules, CA, USA).

### 4.4. Biotinylation of Surface Proteins

Surface biotinylation of cell proteins was performed as previously described [37,38]. Briefly, SH-SY5Y cells treated with either vehicle or IFNβ for 24 h were incubated for 1 h at 4 °C with the cell-impermeable biotinylating agent sulfosuccinimidyl-6-(biotin-amido) hexanoate (sulpho-NHS-LC-biotin) (Pierce, Rockford, IL, USA). After that, the cells were washed with PBS containing 20 mM glycine and solubilized by incubation in RIPA buffer supplemented with 1% Triton X 100. Cell extracts were centrifuged at 10,000× *g* for 5 min at 4 °C and the supernatants were incubated overnight at 4 °C with streptavidin-conjugated agarose beads. The beads were mixed with sample buffer and incubated for 4 min at 100 °C. The proteins were then analyzed by Western blot.

### 4.5. Westen Blotting

Cell proteins were separated by SDS-polyacrylamide gel electrophoresis and were transferred to polyvinylidene difluoride membranes (Millipore). Membranes were blocked, washed, and incubated overnight at 4 °C with one of the following primary antibodies: PD-L1 (cat. no. 13684, Cell Signaling Technology, Danvers, MA, USA) (1:1000); IKBα (cat. no. 4814, Cell Signaling Technology) (1:1000); cleaved caspase 3 (Asp175) (cat. no. 9664, Cell Signaling Technology) (1:1000); caspase 3 (cat no. 9665, Cell Signaling Technology) (1:1000); cleaved-poly (ADP-ribose) polymerase (PARP) (Asp214) (cat. no. 5625, Cell Signaling Technology) (1:1000); PARP (cat. no. 9542, Cell Signaling Technology) (1:1000); phospho-Tyr701-STAT1 (1:1000) (cat no. ST1P-11A5, Thermo Fisher Scientific, Rockford, IL, USA); anti-STAT1 (1:500) (cat no. sc-592, Santa Cruz Biotechnology, Paso Robles, CA, USA); PKR (1:1000) (cat no. 3072, Cell Signaling Technology); survivin (cat. no. 2808, Cell Signaling Technology); Mcl-1 (1:1000) (sc-819, Santa Cruz Biotechnology); pan cadherin (cat. no. 4073, Cell Signaling Technology) (1:2000); actin (1:3000) (cat no. A2066, Sigma-Aldrich); GAPDH (1:5000) (cat no. 247-002, Synaptic Systems, Gottingen, Germany). Thereafter, the membranes were washed and incubated with an appropriate horseradish peroxidase-conjugated secondary antibody (Santa Cruz Biotechnology). Immunoreactive bands were detected by Clarity Western ECL substrate (Bio-Rad Laboratory, Hercules, CA, USA) and were visualized using an ImageQuant LAS-4000 (GE Healthcare, Little Chalfont, UK). The size of immunoreactive bands was determined by using molecular weight standards detected with an ECL suitable antibody (1:1000) (sc-2035, Santa Cruz Biotechnology). Band densities were determined using NIH ImageJ software (US National Institutes of Health, Bethesda, MA, USA, https://imagej.net/ij/ accessed on 24 March 2024). The optical density of the phosphorylated protein bands was normalized to the density of the corresponding total protein in the same sample. For analysis of caspases and PARP, the formation of the cleaved protein was normalized to the level of the corresponding procaspase or non-cleaved PARP measured in the same sample. For the remaining proteins, the densitometric values were normalized to the levels of either actin or a subcellular fraction marker, as indicated.

### 4.6. Isolation of Cell Nuclei

SH-SY5Y nuclei isolation was performed as previously described [39]. Cells were grown in Petri dishes. After drug treatment, cells were washed with ice-cold PBS (pH 7.4) and scraped in an ice-cold lysis buffer. Cell lysates were subjected to centrifugation at 3000× *g* for 10 min at 4 °C while the supernatant was collected and centrifuged at 24,000× *g* for 20 min (cytosolic fraction). The pellets were washed three times in ice-cold washing buffer and layered over a cushion of 1 mL of 1 M sucrose buffer. Following centrifugation at 3000× *g* for 10 min at 4 °C, the nuclei were washed, and the proteins were extracted by incubating the nuclei for 30 min in an extraction buffer. Following centrifugation at 24,000× *g* for 10 min at 4 °C, the nuclear extracts were heated at 100 °C with sample buffer.

### 4.7. Transfection of Small Interfering RNA (siRNA)

SH-SY5Y cells were transfected with either Trilencer-27 Universal scrambled negative control siRNA duplex (SR30004) or PPARA (Human)-3 unique 27mer siRNA duplexes (SR303653) using Lipofectamine 2000 (Invitrogen-Thermo Fisher Scientific, Rockford, IL, USA) as a transfection reagent. Cells grown in 6-well plates were incubated in an antibiotic-free medium for 24 h. The medium was renewed, and the cells were incubated with siRNA duplexes for 4–5 h at 37 °C. Thereafter, the medium was replaced by the growth medium, and the cells were analyzed 48 h post-transfection.

### 4.8. Determination of Cell Count and Viability

A Muse™ Cell Analyzer was used for determining the count and viability of cellular samples using the Muse™ Viability assay kit as instructed by the manufacturer (Millipore Corporation, Merck Life Sciences, Darmstadt, Germany). Briefly, SH-SY5Y cells were treated with OEA or PEA in the presence or absence of IFNβ and were incubated at 37 °C for 24 h. Cells were detached and centrifuged at 300× *g* for 5 min. Finally, the obtained cell pellet was suspended in a complete medium. An amount of 20 µL of this cell suspension was mixed with 380 µL of Count & Viability reagent. The suspension was then kept for 5 min at room temperature and thereafter examined for cell count and viability by the Muse™ Cell Analyzer.

### 4.9. MTT Assay

Cell viability was assessed by an MTT assay in a 96-well plate. SH-SY5Y cells were treated with either the vehicle, OEA, or PEA at different concentrations for 24 h. Cells were incubated with MTT. The blue formazan product was solubilized by the addition of 10% SDS with 10 mM HCl. Absorbance was measured using a Wallach Victor microplate reader (PerkinElmer, Waltham, MA, USA).

### 4.10. Scratch Wound Healing Assay

SH-SY5Y cells were seeded into 24-well cell culture plates and were allowed to grow to 100% confluence as a monolayer. The monolayer was gently scratched across the center of the well with a sterile pipette tip. After scratching, the medium was removed, and the wells were washed twice in PBS solution. Fresh medium containing no FBS and designated treatments were added to each well. Images were obtained from the same fields immediately after scratching (t0) and 48 h later. The scratch was visualized by phase-contrast light microscopy using an Olympus IX51 inverted microscope (Olympus Optical Co., Hamburg, Germany). The images were acquired in randomly selected fields by using an Olympus digital camera and analyzed by ImageJ software (National Institutes of Health, Bethesda, MD, USA). The percentage of the closure of the scratch was then calculated.

### 4.11. Clonogenic Assay

Single-cell suspensions of exponentially growing cultures were seeded into six-well plates in a range of 200 cells/well and were allowed to adhere for 24 h. Cells were then incubated at 37 °C for 10 days. The culture medium was changed every 2 days. At the end of the 10 days, cell growth of all six-well plates was stopped simultaneously. Colonies were fixed with 100% ethanol and were stained with 0.5% crystal violet. A cell colony was defined as a group formation of at least 50 cells and was counted using the Image J software.

### 4.12. RealTime-Glo MT Cell Viability Assay

Luminescence analysis using the RealTime-Glo MT assay kit (Promega, Madison, WI, USA) was used to determine cell viability. Cells grown in 96-well plates (ViewPlate, PerkinElmer) were exposed to the test agents and then incubated with the reagents provided by the kit following the manufacturer’s instructions. The intensity of the luminescent signal generated in viable cells was measured by a Wallac Victor III microplate reader (PerkinElmer, Waltham, MA, USA). Assays were performed in triplicate.

### 4.13. Caspase-Glo 3/7 Assay

The cells that were grown in 96-well plates (ViewPlate-96) were incubated as specified in the text. The cells were then assayed for caspase activity by using the Caspase-Glo 3/7 assay kit (Promega, Madison, USA), according to the manufacturer’s instructions. Luminescence intensity was measured by using a Wallac Victor III microplate reader (PerkinElmer, Waltham, MA, USA). The assays were performed in triplicate.

## 5. Statistical Analysis

The results were reported as mean ± SEM. Statistical analysis was performed using Graph Pad Prism (San Diego, CA, USA, https://www.graphpad.com/ accessed on 24 March 2024). The control group or IFNβ was set as 100% or 1 with a variance obtained by expressing each control value as a percentage of the mean of the raw values of the control group or IFNβ, which was included in each independent experiment. Unpaired Student’s *t*-tests and analysis of variance (ANOVA) followed by Tukey’s post hoc test were performed, as appropriate, to assess significant differences between the experimental groups. A value of *p* < 0.05 was considered to be statistically significant.

## 6. Conclusions

So far, lipids have been linked to several conditions including diabetes, atherosclerosis, cancer, inflammation, and multiple sclerosis. To date, limited data are available on the role played by these molecules during inflammation, specifically in cancerous cells. In this regard, we aimed to evaluate the effect of the exogenous administration of OEA and PEA in combination with the cytokine IFNβ on apoptosis using the human neuroblastoma SH-SY5Y cell line. In our study, we demonstrated that OEA and PEA empower the apoptotic response mediated by IFNβ through independent signaling pathways and potentiate the cell death induced by this cytokine in SH-SY5Y cells. Moreover, these lipids also increased the IFNβ-mediated induction of PD-L1 and the phosphorylation of p38 MAPK, indicating that the synergic effect of OEA and PEA with IFNβ might be valid beyond apoptosis. More investigations are required to understand the effects of OEA and PEA combined with IFNβ on different human neuroblastoma cell lines; nevertheless, our study offers substantial and novel data emphasizing the promising use of PPARα receptor agonists as possible new therapeutic targets to accompany, in the future, classical cancer therapy.

## Data Availability

Data are contained within the article and Supplementary Materials.

## Supplementary Materials

The following supporting information can be downloaded at: https://www.mdpi.com/article/10.3390/molecules29071592/s1, Figure S1: Cells were exposed for 6 h to OEA and PEA (3 μM) and then to IFNβ (5 ng/mL) for 12 h and were subjected to caspase 3/7 activity. The values are expressed as percent of control (vehicle). * *p* < 0.05 and ** *p* < 0.01 versus vehicle-treated cells. The results are represented as mean ± SEM of four independent experiments.
