# Supplementary material for: Oleoylethanolamide and Palmitoylethanolamide Enhance IFNβ-Induced Apoptosis in Human Neuroblastoma SH-SY5Y Cells
Source: Molecules. 2024 Apr 2;29(7):1592. doi: 10.3390/molecules29071592 (PMC11013881; doi:10.3390/molecules29071592)
Supplement: Supplementary file 1 [file molecules-29-01592-s001.zip › molecules-2912457-supplementary.pdf]

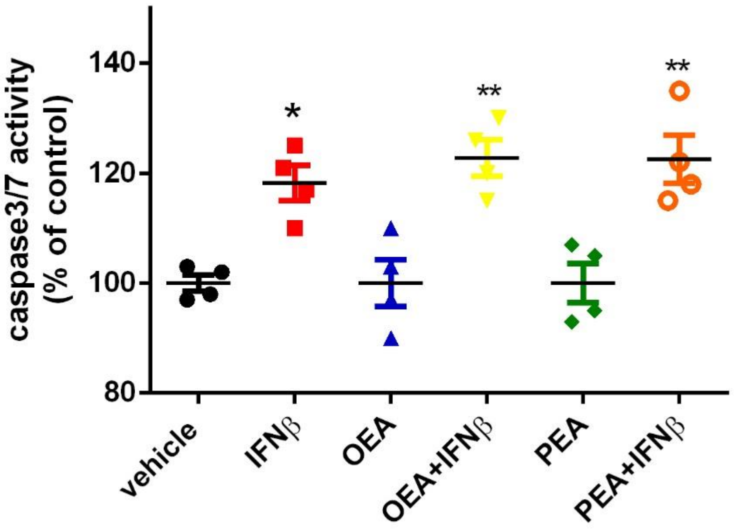

Figure S1. Cells were exposed for 6 hours to OEA and PEA (3 µM) and then to IFNβ (5 ng/ml) for 12 hours and were subjected to caspase 3/7 activity. The values are expressed as percent of control (vehicle). *p< 0.05 and **p< 0.01 *versus* vehicle-treated cells . The results are represented as mean ± SEM of four independent experiments.
